# Magnesium Limitation Is an Environmental Trigger of the *Pseudomonas aeruginosa* Biofilm Lifestyle

**DOI:** 10.1371/journal.pone.0023307

**Published:** 2011-08-16

**Authors:** Heidi Mulcahy, Shawn Lewenza

**Affiliations:** Department of Microbiology, Immunology and Infectious Diseases, University of Calgary, Calgary, Canada; Charité-University Medicine Berlin, Germany

## Abstract

Biofilm formation is a conserved strategy for long-term bacterial survival in nature and during infections. Biofilms are multicellular aggregates of cells enmeshed in an extracellular matrix. The RetS, GacS and LadS sensors control the switch from a planktonic to a biofilm mode of growth in *Pseudomonas aeruginosa*. Here we detail our approach to identify environmental triggers of biofilm formation by investigating environmental conditions that repress expression of the biofilm repressor RetS. Mg^2+^ limitation repressed the expression of *retS* leading to increased aggregation, exopolysaccharide (EPS) production and biofilm formation. Repression of *retS* expression under Mg^2+^ limitation corresponded with induced expression of the GacA-controlled small regulatory RNAs *rsmZ* and *rsmY* and the EPS biosynthesis operons *pel* and *psl*. We recently demonstrated that extracellular DNA sequesters Mg^2+^ cations and activates the cation-sensing PhoPQ two-component system, which leads to increased antimicrobial peptide resistance in biofilms. Here we show that exogenous DNA and EDTA, through their ability to chelate Mg^2+^, promoted biofilm formation. The repression of *retS* in low Mg^2+^ was directly controlled by PhoPQ. PhoP also directly controlled expression of *rsmZ* but not *rsmY* suggesting that PhoPQ controls the equilibrium of the small regulatory RNAs and thus fine-tunes the expression of genes in the RetS pathway. In summary, Mg^2+^ limitation is a biologically relevant environmental condition and the first bonafide environmental signal identified that results in transcriptional repression of *retS* and promotes *P. aeruginosa* biofilm formation.

## Introduction

In the natural environment and during infection of susceptible hosts bacteria predominantly grow as biofilms. Biofilms are surface-associated, microbial communities, which are embedded in an extracellular matrix composed primarily of bacterial-derived exopolysaccharides (EPS) and DNA [1]–[3]. Biofilms have been intensively studied in recent years due to their significance in industrial, natural and medical settings. A multicellular biofilm lifestyle for bacteria has survival advantages compared to bacteria living as planktonic individual cells. During infection, growth as a biofilm confers resistance to multiple environmental stresses, antibiotics and the immune system [4]–[6].

The environmental bacterium and opportunistic human pathogen *Pseudomonas aeruginosa* PAO1 is one of the most widely used model organisms for studying bacterial biofilm formation. *P. aeruginosa* is a versatile Gram-negative bacterium that grows in soil and marine environments, as well as on plant and animal tissues [7]. *P. aeruginosa* has also emerged as a major opportunistic human pathogen during the past century [8] and individuals with the genetic disease Cystic Fibrosis (CF) are particularly susceptible [9]. A large body of *in vitro* and *in vivo* data in the literature indicates that *P. aeruginosa* exists as a biofilm in the lungs of CF patients [10]–[15].

Biofilm formation protects bacteria from harsh and stressful conditions [16] and the ability of bacteria to adapt to changing environmental conditions is essential for survival. *P. aeruginosa* is capable of sensing environmental conditions and adapting to changing conditions through modification of gene expression. A common mechanism of adaptation, which is both rapid and reversible, utilizes two-component systems (TCS) [17]. In many bacterial systems, TCS are involved in the regulation of biofilm formation. Typical TCS are comprised of a membrane-anchored histidine kinase sensor and a cytoplasmic response regulator (RR). After the sensor detects specific environmental signals, a signal transduction cascade is initiated that results in phosphorylation of a RR, which activates or represses necessary target genes. In *P. aeruginosa* multiple sensor proteins have been identified, mostly through genetic screens, which are important for *P. aeruginosa* biofilm formation. These include the sensor proteins GacS, RetS, LadS, SadS and PhoQ [18]–[23] as well as the BfiS, BfmS and MifS sensors, which control progression through distinct stages of biofilm maturation [24]. However, the exact signals detected by most of these environmental sensors are unknown.

RetS is required for expression of the virulence-associated type III secretion system (T3SS) and for repression of the *pel* and *psl* biofilm matrix EPS biosynthesis genes through both transcriptional and post-transcriptional regulation in *P. aeruginosa* [19], [20], [25], [26]. RetS directly interacts and forms heterodimers with the GacS sensor protein, preventing activation of the GacAS pathway and repressing biofilm formation [27], [28]. Reduced levels of RetS favors the formation of GacS homodimers, autophosphorylation of GacS and activation of the GacA-controlled the small regulatory RNAs (sRNA), *rsmZ* and *rsmY* [29], [30]. *RsmZ* and *rsmY* bind and sequester the post-transcriptional regulatory protein RsmA, which normally functions to bind other target mRNAs. RsmA-mediated regulation can be direct, through mRNA binding and preventing initiation of translation such as that observed for *psl* [26] or indirect, by interfering with the translation of specific regulatory factors [25]. However in addition post-transcriptional regulation of *psl* genes, genome-wide transcriptional profiling identified *retS* as a transcriptional regulator of the *pel* and *psl* EPS operons [19].

It has been proposed that the RetS sensor responds to environmental conditions encountered during acute infections maintaining *P. aeruginosa* in a planktonic growth state capable of Type III secretion [20]. In contrast to this model, we report here that Mg^2+^ limitation causes transcriptional repression of *retS* and promotes a switch from the planktonic to a biofilm lifestyle. Repression of *retS* occurred through direct repression by the cation sensing PhoPQ two-component system. Recent work from our laboratory has identified extracellular DNA as a chelator of divalent cations that activates the PhoPQ two-component system, resulting in the expression of antibiotic resistance genes [31]. To our knowledge this is the first demonstration of a specific environmental signal that promotes a switch to a biofilm mode of growth by acting through the RetS/LadS/GacS pathway. As DNA is abundant both in the natural and host environment [32]–[37], Mg^2+^ limitation is a relevant biological signal and is encountered ubiquitously by *P. aeruginosa*.

## Results

### Identification of Mg^2+^ limitation as an environmental signal that represses *retS* expression

RetS is required for repression of EPS biosynthesis genes and preventing biofilm formation in *P. aeruginosa* [19]. Our strategy was to identify environmental signals that repressed the expression of *retS* and thus likely promoted biofilm formation in *P. aeruginosa*. To identify environmental signals that repressed the levels of RetS, we monitored expression of the *retS* promoter fused to the *lux* (bioluminescence) reporter in diverse growth conditions. The growth conditions tested were intended to mimic the conditions faced by *P. aeruginosa* during chronic lung infections (Table S1).

**Figure 1:**
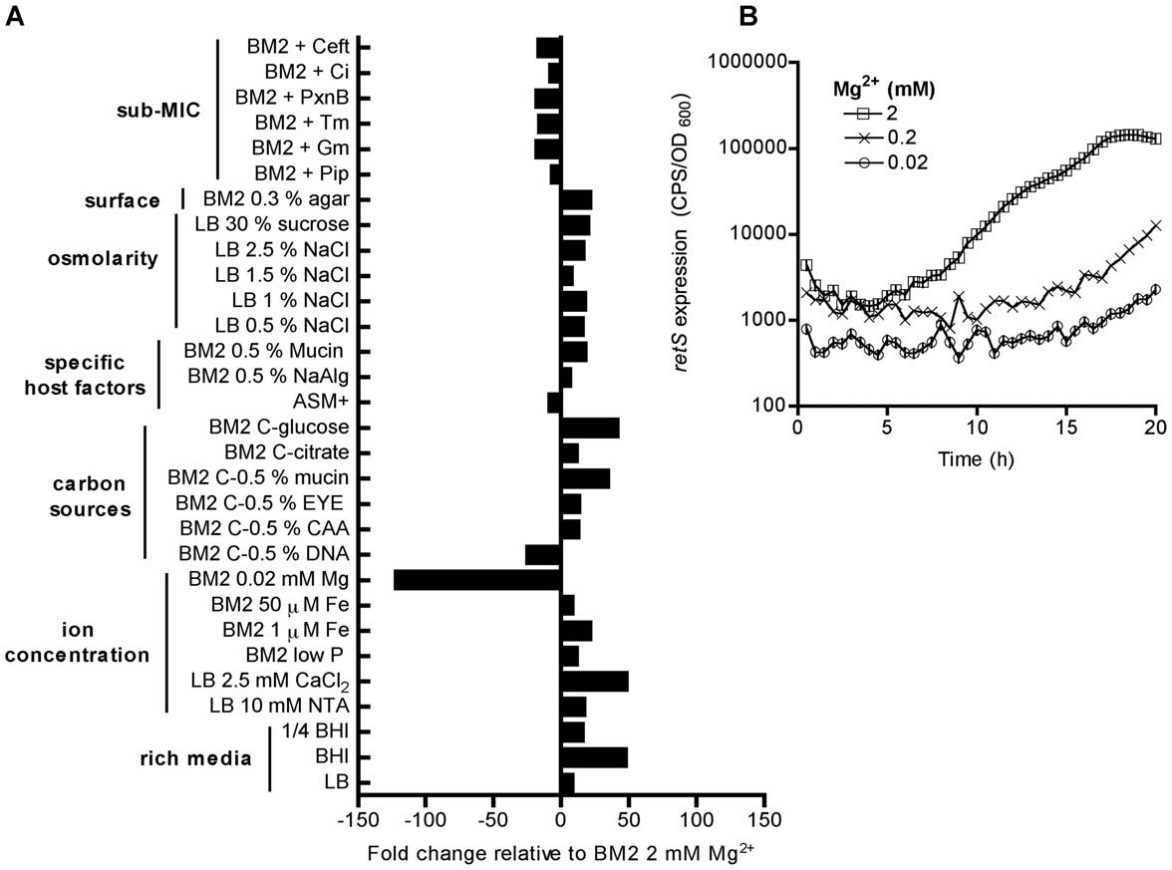
Limiting Mg^2+^ repressed *retS* expression. (A) The expression of *retS* was monitored in PAO1, using a promoter *lux* fusion (*pMS402-lux*), in different media and expressed as fold change in the medium indicated relative to BM2 2 mM Mg^2+^ during late log phase (11 h). (B) Expression of *retS* (CPS/OD_600_) was repressed by Mg^2+^ limitation in a concentration-dependent manner. Values are representative of at least 3 independent experiments. For each experiment the standard deviations were not greater that +/−10% of the mean value.

RetS expression was induced between 10- and 50-fold, or repressed between 7- and 125-fold in the conditions tested. Over the course of 20 h-growth, maximal repression (up to 125-fold) of *retS* was observed in limiting Mg^2+^ conditions (Figure 1A). We were particularly interested in *retS* gene expression under limiting Mg^2+^ growth conditions because we recently showed that extracellular DNA is an efficient chelator of divalent cations including Mg^2+^ [31]. Consistent with DNA acting as a chelator of Mg^2+^, *retS* expression was repressed up to 25-fold in media supplemented with exogenous DNA (Figure 1A). The expression of *retS* was repressed in a Mg^2+^ concentration-dependent manner (Figure 1B), with maximal repression observed at 0.02 mM Mg^2+^. Gene expression was normalized to growth as growth rates of cells grown under 2 mM or 0.02 mM Mg^2+^ were similar (6.2 h and 6.6 h, respectively).

### Mg^2+^ limitation results in a gene expression signature consistent with the switch to a biofilm lifestyle

Several studies examining the regulation of virulence factor production in *P. aeruginosa* identified a potential regulatory switch involving the RetS/LadS/GacS two component sensors, that controls the transition between acute and chronic infection related phenotypes [19], [20]. Identification of limiting Mg^2+^ as an environmental signal that represses expression of the known biofilm repressor gene *retS* prompted us to investigate the expression of a panel of genes controlled by the RetS/LadS/GacS pathway that are known to be important for either acute or chronic *P. aeruginosa* infections.

**Figure 2:**
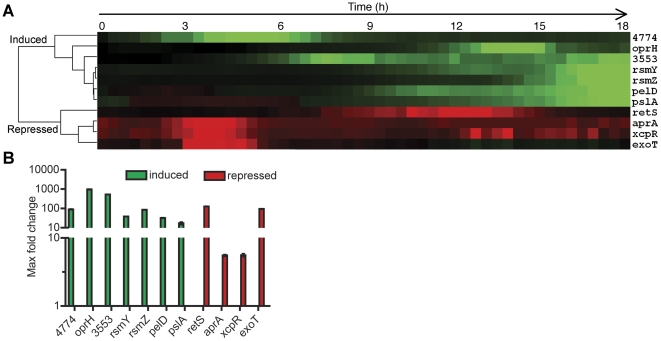
Limiting Mg^2+^ induced expression of EPS biosynthesis and small regulatory RNAs while *retS* expression was repressed. (A) Gene expression data are expressed as fold induction (green) or fold repression (red) of bacteria cultured in BM2 0.02 mM Mg^2+^ relative to BM2 2 mM Mg^2+^. For each gene, each square represents relative expression values measured at 20 min intervals throughout growth. Gene expression profiles were grouped by hierarchical clustering using average linkage analysis (Cluster 3.0) and visualized using Treeview. (B) Maximal fold change in gene expression of transcriptional *lux* fusions in BM2 0.02 mM Mg^2+^ relative to BM2 2 mM Mg^2+^ over 18 h. Values are representative of at least 3 independent experiments and error bars represent the standard error of the mean (SEM).

The data in Figure 2A represents the time course of fold induction (green) or repression (red) of genes in BM2 growth medium with limiting Mg^2+^ relative to high Mg^2+^ over 18 h of growth. In the cluster analysis depicted in Figure 2A, gene expression profiles cluster into two distinct groups: genes that are upregulated or downregulated in limiting Mg^2+^ conditions. The cluster of induced genes included genes previously identified as regulated by Mg^2+^ limitation by the PhoPQ or PmrAB TCS. These included *PA3553 (arnC)* from the LPS modification operon *PA3552-3559*, the outer membrane protein *oprH*, and a putative polyamine synthesis gene *PA4774* [38]–[40]. Additionally the small regulatory RNAs encoded by *rsmZ* and *rsmY*, as well as genes from two independent EPS biosynthesis operons, *pelD* and *pslA*, were also induced by Mg^2+^ limitation (Figure 2). In contrast, genes encoding the *retS* biofilm regulator, type II secretion system genes (*xcpR* and *aprA*) and a type III secretion system effector, *exoT*, clustered together as genes repressed by Mg^2+^ limitation. To demonstrate the magnitude of induction or repression, Figure 2B illustrates the maximal change in gene expression over 18 h.

According to the current model of reciprocal regulation of acute and chronic infection related traits by the RetS/LadS/GacS pathway, biofilms and chronic infections are promoted under conditions that induce EPS expression while simultaneously repressing the T2SS and T3SS [19], [20]. The expression profiles observed for the genes shown in Figure 2 are consistent with limiting Mg^2+^ acting as a signal that promotes a switch from the planktonic to an aggregative, biofilm mode of growth. We observed that maximal repression of the T2SS and T3SS genes occurred in the log phase of growth, prior to maximal repression of *retS*. This indicates that additional factors, the identification of which are beyond the scope of this study, may be involved in regulating these secretion system genes under Mg^2+^ limitation.

### Mg^2+^ limitation promotes biofilm formation and aggregation

**Figure 3:**
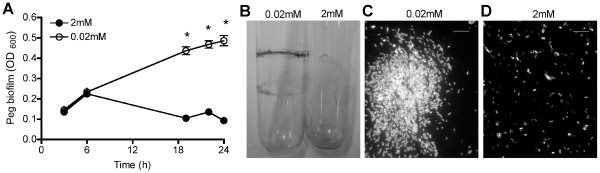
Mg^2+^ limitation promoted biofilm formation and aggregation in *P. aeruginosa*. (A) Attachment of cells to polystyrene pegs was monitored using crystal violet staining over time. Each point represents the average value obtained from twelve pegs and error bars represent the standard deviation. *, significant increase (p<0.05, ANOVA) in BM2 0.02 mM Mg^2+^ compared to that of BM2 2 mM Mg^2+^. (B) Attachment of cells to glass after 24 h measured using crystal violet staining. (C–D) Aggregation of PAO1 pCHAP6656 in liquid cultures was visualized by fluorescence microscopy in BM2 (C) 0.02 mM or (D) 2 mM Mg^2+^. Images are representative of data obtained in 3 independent experiments. Scale bars represent 10 µM.

Pel and Psl EPS are essential biofilm matrix components that are important for adhesion and biofilm formation [41], [42]. As the expression of *pel* and *psl* EPS biosynthesis genes were strongly induced by Mg^2+^ limitation, we predicted that biofilm formation would be increased under these conditions. Mg^2+^ limitation strongly promoted biofilm formation, as measured by crystal violet (CV) staining of the total biomass adhered to the polystyrene pegs (Figure 3A) or to glass surfaces (Figure 3B). Additionally, large bacterial aggregates (50–70 µM diameter) (Figure 3C) were observed in mid-log phase planktonic cultures under Mg^2+^ limitation. No large aggregates of bacteria were visible when grown in high Mg^2+^ (Figure 3D).

### The cation chelators DNA and EDTA induce biofilm formation

We have recently shown that extracellular DNA can function as a cation chelator that activates the cation-sensing PhoPQ TCS, leading to increased antimicrobial peptide resistance [31]. Since limiting Mg^2+^ is an environmental condition that also promoted biofilm formation, we proposed that cation chelation by extracellular DNA may ultimately impose a cation limitation on cells and promote biofilm formation.

**Figure 4:**
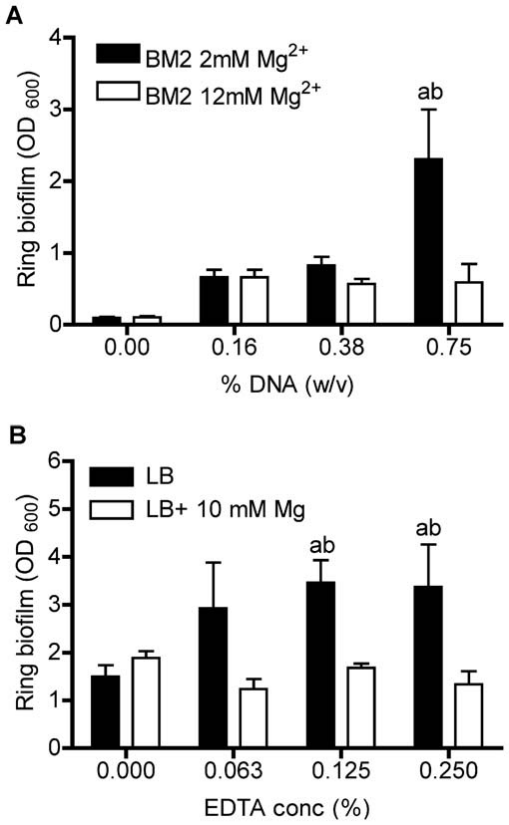
Mg^2+^ chelation by DNA and EDTA induced biofilm formation. Attachment of PAO1 to wells in a 96-well plate was assessed using crystal violet staining after 24 h growth in (A) BM2 2 or 12 mM Mg^2+^ with increasing concentration of salmon sperm DNA or (B) LB in the absence and presence of 10 mM Mg^2+^ with increasing concentrations of EDTA. Values are representative of at least 3 independent experiments and error bars represent the standard error of the mean (SEM). a, significant increase (p<0.05, ANOVA) in media alone compared to that of media with DNA or EDTA; b, significant increase (p<0.05, ANOVA) in media with DNA or EDTA compared to that of media with DNA or EDTA and excess (10 or 12 mM) Mg^2+^.

To test this hypothesis, we cultivated ring biofilms in media with increasing concentrations of exogenous DNA. The addition of salmon sperm DNA promoted biofilm formation in a concentration-dependent manner (Figure 4A). To confirm that the chelating activity of DNA promoted biofilm formation, as opposed to the adhesive capacity of DNA, exogenous Mg^2+^ was added to cultures. The biofilm promoting effect of exogenous DNA was significantly reduced by addition of 10 mM excess Mg^2+^, to a final concentration of 12 mM Mg^2+^(Figure 4A). The cation chelator EDTA also caused a concentration-dependent increase in biofilm formation that was also completely neutralized by 10 mM excess Mg^2+^ (Figure 4B).

### Mg^2+^ limitation induces production of EPS in the biofilm matrix

**Figure 5:**
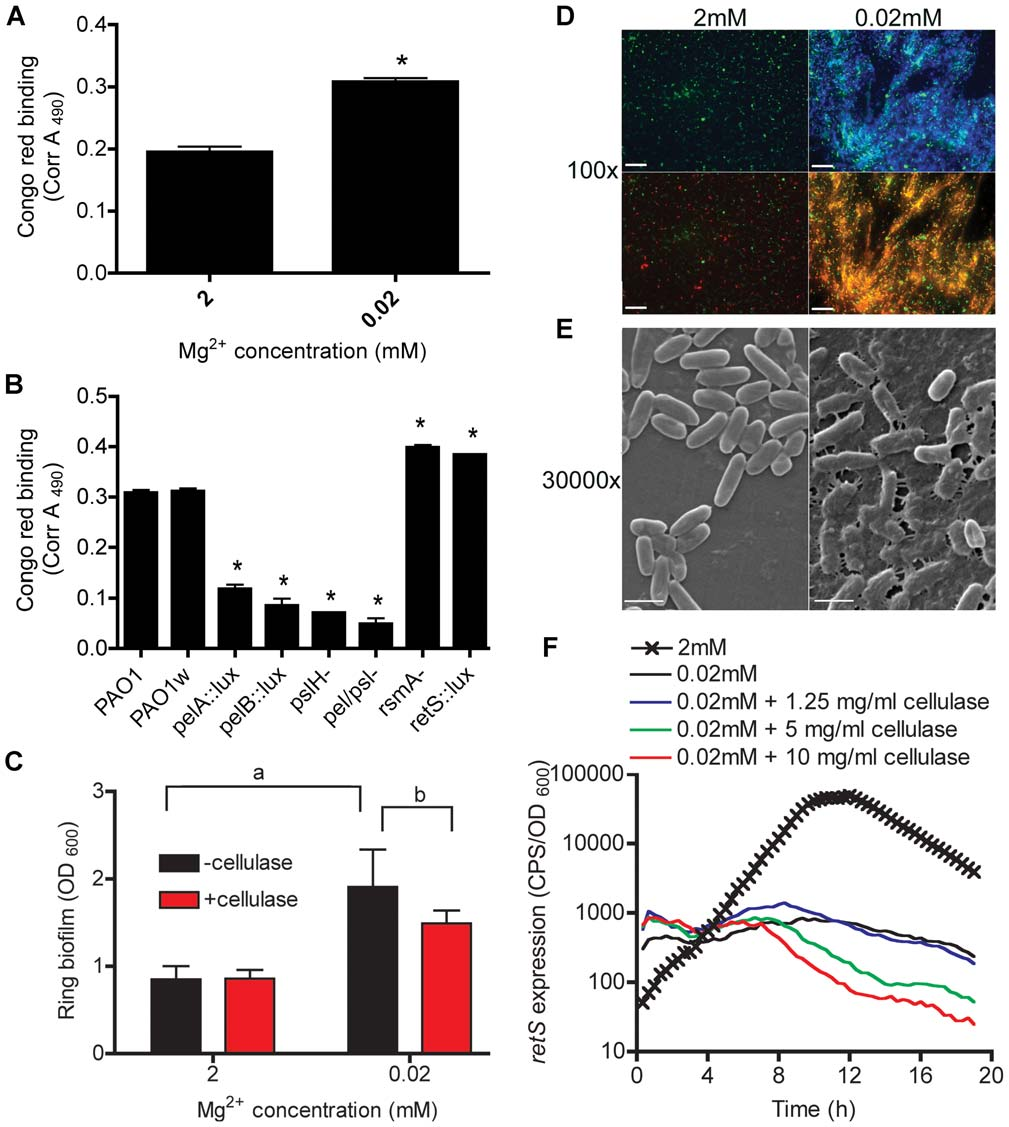
Mg^2+^ limitation induced EPS production. EPS production was quantified after 24 h growth of (A) PAO1 in BM2 2 or 0.02 mM Mg^2+^ or (B) Wild type and mutant strains grown in BM2 0.02 mM Mg^2+^ using congo red binding. (C) Ring biofilm formation in 96 well plates was quantitated in BM2 2 mM Mg^2+^ or 0.02 mM Mg^2+^ in the absence and presence of 5 mg/ml of cellulase. Values are representative of at least 3 independent experiments and error bars represent the standard error of the mean (SEM). (D) Fluorescence microscopy was used to visualize aggregation and EPS production. Bacteria were grown in BM2 liquid cultures with 2 or 0.02 mM Mg^2+^ supplemented with 200 µg/ml calcofluor. At 24 h cells were stained with 1 µM SYTO9 (live cells, green, upper panel) and 10 µM propidium iodide (dead cells and DNA, red, lower panel). Scale bars represent 10 µM. (E) For scanning electron microscopy, PAO1 was grown as biofilms on polystyrene pegs in BM2 containing 2 or 0.02 mM Mg^2+^ for 48 h. Scale bars represent 2 µM. Images are representative of data obtained in three independent experiments. (F) Expression of *retS*, measured using a promoter *lux* fusion (*pMS402-lux*), in BM2 2 mM Mg^2+^ or 0.02 mM Mg^2+^ in the absence and presence of EPS-degrading cellulase. Values are representative of at least 3 independent experiments. For each experiment the standard deviations were not greater that +/−10% of the mean value. *, significant difference (p<0.05, ANOVA) relative to control conditions or between wildtype and mutant strains. a, significant increase (p<0.05, ANOVA) in BM2 0.02 mM Mg^2+^ compared to that of BM2 2 mM Mg^2+^; b, significant difference (p<0.05, ANOVA) in BM2 0.02 mM compared to that of BM2 0.02 mM with 5 mg/ml of cellulase.

Biofilm formation is induced under limiting Mg^2+^ conditions (Figure 3). To confirm increased EPS production under limiting Mg^2+^ conditions, we compared EPS production in planktonic cultures grown in 0.02 and 2 mM Mg^2+^. Using the congo red assay for measuring EPS production [43], *P. aeruginosa* produced significantly more total EPS under Mg^2+^ limiting conditions relative to high Mg^2+^ conditions (Figure 5A). Single mutants in either the *pel* or *psl* EPS biosynthesis operons showed significantly decreased congo red binding, 40% of wild-type levels (Figure 5B). Furthermore, the double *pel*/*psl* mutant exhibited 60% reduced congo red binding relative to PAO1, confirming the requirement of Pel and Psl for EPS production (Figure 5B). Both *rsmA* and *retS* mutants (PAZH13, *retS::lux*) exhibited elevated EPS levels and were included as positive controls for EPS production.

Cellulase has been used previously to degrade Psl EPS and effectively reduces *P. aeruginosa* biofilm formation [43]. The addition of cellulase during biofilm cultivation had no effect on biofilms cultivated in high Mg^2+^ conditions but caused a significant reduction in biofilm formation under Mg^2+^ limitation (Figure 5C). This data indicates the importance of EPS in biofilm formation under limiting Mg^2+^ conditions as degradation of the EPS matrix with cellulase results in significantly lower biofilm formation during growth in limiting Mg^2+^.

To correlate EPS production with surface attachment, we tested biofilm formation phenotypes of EPS synthesis mutants. Individual *pel* or *psl* and double *pel/psl* mutants exhibited significantly reduced biofilm formation (up to 80%) relative to PAO1 (Figure S1). Additionally, we found that biofilm formation under Mg^2+^ limitation was not significantly reduced in mutants lacking type IV pili or flagella production (Figure S1). This suggests that Mg^2+^-limited biofilm formation does not require the presence of pili or flagella, which are generally important adhesins for biofilm formation [44].

Calcofluor binds sugars with β-1,4 linkages and has previously been shown to positively correlate with EPS production in a number of bacterial species including *P. aeruginosa* [41], [43], [45], *Salmonella enterica* serovar Typhimurium, and *Escherichia coli* [46]–[49]. Microscopic analysis indicated calcofluor bound to *P. aeruginosa* aggregates cultured under limiting Mg^2+^ and the absence of both aggregation and calcofluor binding in high Mg^2+^ conditions (Figure 5D). Aggregates formed under limiting Mg^2+^-conditions were EPS-dependent as *pelD*, *pslH* and *pel/psl* mutants failed to aggregate or stain positively for EPS under these conditions (Figure S2). Scanning electron microscopy indicated that biofilms grown under high Mg^2+^ conditions resulted in diffuse cell clusters with no observable interconnecting matrix or conditioning layer (Figure 5E). However, biofilms grown in limiting Mg^2+^ conditions clearly produced an extracellular matrix that coated the plastic surface and connected cells in web-like patterns (Figure 5E). Taken together a combination of phenotypic assays and microscopic analysis confirmed EPS overproduction in limiting Mg^2+^ conditions and that increased expression of EPS promoted biofilm formation in limiting Mg^2+^ conditions.

### Aggregation is not the signal that leads to *retS* repression under Mg^2+^ limiting conditions

Our data was consistent with the hypothesis that Mg^2+^ limitation led to increased EPS production and biofilm formation, as a result of repression of the biofilm repressor RetS. An alternative interpretation to this data could be that aggregation itself may serve as the environmental cue for repressing *retS* transcription. To test this possibility, we examined the effects of adding cellulase, which degrades EPS, on *retS* expression. Cellulase was added to limiting Mg^2+^ cultures at the beginning of growth, as cellulase is known to degrade the EPS matrix and reduce cell-cell aggregation [43] and biofilm formation (Figure 5C). Cellulase treatment did not result in an increase in *retS* expression (Figure 5F). Similarly, *retS* expression was still repressed in the non-aggregating *pel/psl* double mutant strain compared to wildtype levels (data not shown) indicating that Mg^2+^ limitation represses *retS* expression independent of aggregation.

### Overexpression of the RetS sensor prevents biofilm formation under Mg^2+^ limitation

**Figure 6:**
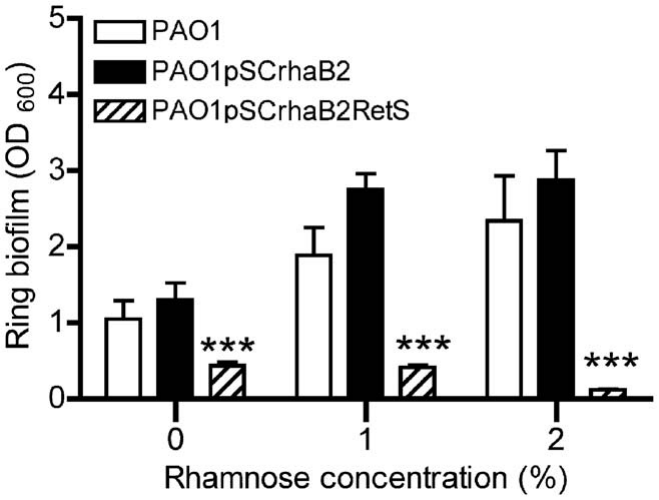
Rhamnose-inducible expression of RetS prevented biofilm formation in Mg^2+^ limiting growth conditions. Crystal violet staining of ring biofilms was performed in biofilms grown in BM2 0.02 mM Mg^2+^ with increasing rhamnose concentrations, where *retS* expression was induced using pSCRhaRetS. Values are representative of at least 3 independent experiments and error bars represent the standard error of the mean (SEM). ***, significant difference (p<0.001, ANOVA) in PAO1 or PAO1pSCRhaB2 (vector control) compared to that of PAO1pSCRhaRetS.

Previous studies showed mutation of *retS* results in a hyperbiofilm phenotype due to hyperproduction of Pel and Psl exopolysaccharides [19], [20]. The expression of *retS* is repressed in Mg^2+^ limitation, which correlates with increased EPS production and biofilm formation. To definitively show that reduced levels of *retS* expression under Mg^2+^ limitation was essential for promoting biofilm formation, the *retS* gene was cloned (without its native promoter) under the control of a rhamnose-inducible promoter [50]. Biofilms were cultivated in the presence of increasing amounts of rhamnose to induce *retS* expression in a concentration-dependent manner. In the absence of rhamnose, expression of RetS from pSCRhaB2RetS caused a reduction in biofilm formation, most likely due to low, basal levels of expression of RetS from the plasmid (Figure 6). In support of our hypothesis, rhamnose-induced expression of RetS from pSCRhaB2RetS inhibited biofilm formation in limiting Mg^2+^ conditions (Figure 6), a condition which otherwise promotes biofilm formation (Figure 3), as a result of reduced *retS* expression (Figure 1).

### The Mg^2+^ sensing PhoPQ TCS influences the RetS regulatory pathway

Our results above identified a novel environmental signal that induced biofilm formation through repression of RetS. RetS acts as part of a complex signaling pathway that reciprocally regulates the expression of numerous virulence related genes [19]. It is unlikely that RetS itself responds to Mg^2+^ levels, since RetS is predicted to bind carbohydrates [51], [52]. Furthermore as PhoPQ is the only known TCS that is capable of sensing and responding to cation limitation in *P. aeruginosa* [53], [54], we hypothesized that PhoPQ repressed *retS* expression, and thus influenced the RetS/GacS/LadS pathway. If this model was true, it would be predicted that the expression profile of genes in the RetS pathway would be reversed in a *phoP::xylE* mutant relative to PAO1, and that high levels of *retS* expression in *phoP::xylE* would correspond to decreased levels of the small regulatory RNAs *rsmZ and rsmY* and the *pel* and *psl* EPS biosynthesis genes.

**Figure 7:**
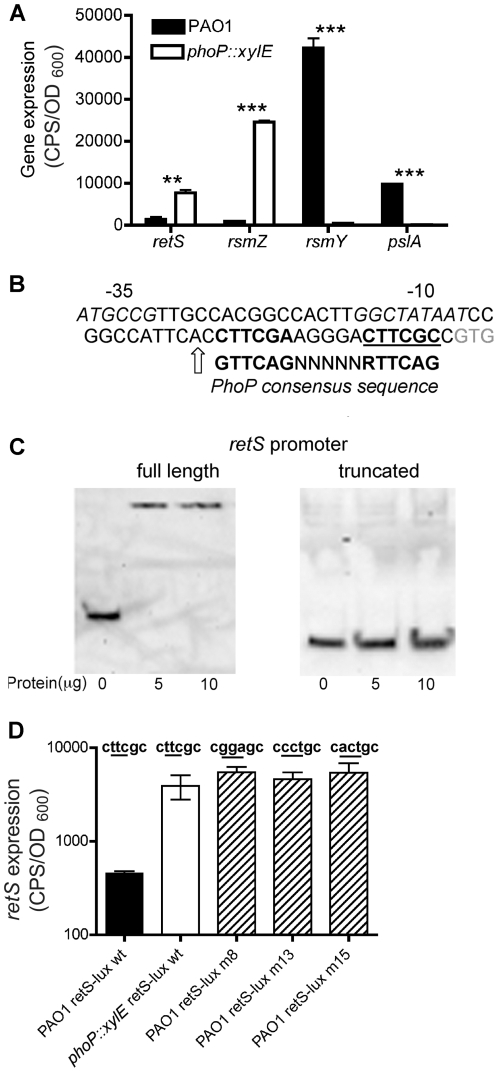
Crosstalk between the Mg^2+^ sensing PhoPQ TCS and the RetS biofilm regulatory pathway. (A) PAO1 and *phoP::xylE* were cultured in BM2 0.02 mM Mg^2+^. Expression of *retS*, *rsmZ*, *rsmY* and *psl* was analysed using plasmid encoded promoter-*lux* fusions. Gene expression data during log phase are shown. Values are representative of at least 3 independent experiments and error bars represent the standard error of the mean (SEM). (B) Identification of putative PhoP box (repeats indicated in bold) in the *retS* promoter with the predicted (BPROM) −10 and −35 sites (italicized). A full length (366 bp) and truncated (239 bp) *retS* promoter, with and without the predicted PhoP box (bold) (genome coordinates 5451891–5452257 and 5451891–5452131, respectively) were PCR amplified and labeled with DIG. Arrow indicates last nucleotide of reverse primer for truncated promoter, upstream of the GTG start codon (grey). (C) Gel shift assays of DIG-labeled full length and truncated *retS* promoter fragments with purified His_6_-PhoP protein. (D) *RetS* promoter activity (*lux*) in BM2 0.02 mM Mg^2+^ cultures of PAO1 and *phoP::xylE* using wildtype *retS* promoter sequences and in PAO1 following mutation (underlined) of the second repeat in the PhoP box in the *retS* promoter. Data shown are representative of 3 independent experiments and error bars represent the standard error of the mean (SEM). **, significant difference (p<0.01, ANOVA); ***, significant difference (p<0.001, ANOVA) in PAO1 compared to that of *phoP::xylE*.

Expression of *retS* was derepressed in a *phoP::xylE* mutant compared to PAO1 in BM2 0.02 mM Mg^2+^, indicating that PhoP is required to repress *retS* (Figure 7A). No differences in *retS* expression were observed between PAO1 and *phoP::xylE* in BM2 2 mM Mg^2+^ (data not shown), a condition where the PhoPQ system is inactive. Expression of the *psl* EPS genes was reduced (60-fold), as predicted, in the *phoP::xylE* mutant (Figure 7A). In support of the link between PhoP and EPS production, a *phoP::xylE* mutant produced significantly less EPS as measured by the congo red binding assay and by direct visualization using transmission electron microscopy (Figure S3). Both *rsmY* and *rsmZ* had increased expression under limiting Mg^2+^conditions (Figure 2) and it was hypothesized that levels of both *rsmY* and *rsmZ* would decrease in a *phoP::xylE* mutant. This hypothesis was true for *rsmY* expression, which was repressed in the *phoP::xylE* mutant. In contrast, *rsmZ* expression was more highly expressed in a *phoP::xylE* mutant (Figure 7A). While *rsmY* and *rsmZ* are both induced under limiting Mg^2+^conditions in PAO1 (Figure 2), the relative levels of *rsmY* are higher (45-fold) (Figure 7A). In a *phoP::xylE* mutant this expression pattern is reversed, with higher relative levels of *rsmZ* observed (47-fold) (Figure 7A), indicating that the PhoPQ system differentially regulates *rsmZ* and *rsmY* expression.

### PhoP directly represses *retS* and *rsmZ* expression

We previously characterized the PhoP regulon and identified PhoP binding sites in promoters of genes directly controlled by PhoPQ [40]. We examined the *retS* promoter for the presence of a PhoP consensus binding site (GTTCAGNNNNNRTTCAG) and found a candidate PhoP binding site between the start codon and the −10 promoter region (Figure 7B); a position consistent with PhoP acting as a repressor of gene expression. PhoP binding assays were performed using purified His_6_-PhoP with a 366 bp promoter fragment. PhoP bound this fragment causing a shift (Figure 7C). Using a truncated promoter fragment (255 bp), which excluded the predicted PhoP box, we observed that PhoP did not bind to the *retS* promoter lacking the predicted PhoP binding site (Figure 7C).

To confirm that PhoP repression of *retS* expression required the putative PhoP binding site, we constructed site-directed mutations in the PhoP box within the *retS* promoter. We initially constructed a *retS* promoter fragment that lacked the entire 18 bp PhoP box and this promoter-*lux* fusion had no promoter activity (data not shown), likely as a result of deleting the transcription start site. To ensure the mutation strategy did not interfere with transcription, we created site-directed changes in the second direct repeat of the PhoP box (Figure 7D). Substitution of the wildtype sequence cgttcc for cgggac, cgcctc or cgactc resulted in derepression of *retS* under limiting Mg^2+^ conditions in wildtype PAO1, thus confirming the identity of this sequence as the site of PhoP-mediated repression (Figure 7D).

**Figure 8:**
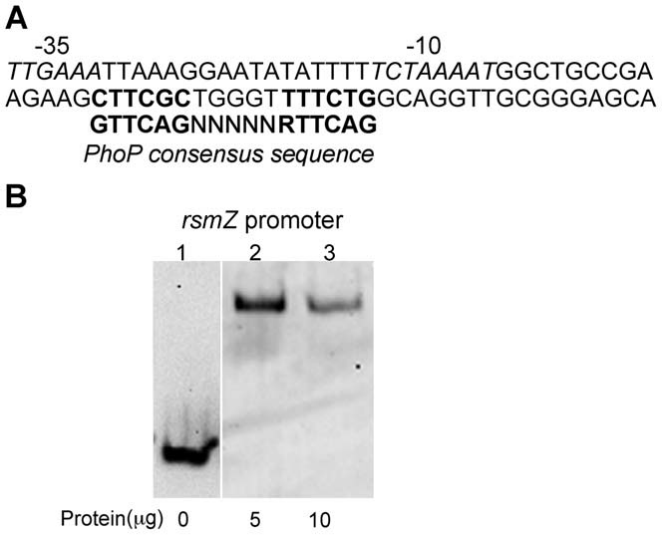
PhoP repressed *rsmZ* expression through direct binding. (A) Identification of putative PhoP box (bold) in *rsmZ* promoter (genome coordinates 4057874–4057855); −10 and −35 indicated in italics. (B) Gel shift assay of DIG-labeled *rsmZ* promoter fragment with purified His_6_-PhoP protein. Data shown are representative of 3 independent experiments.

Our data suggested that PhoP controlled multiple genes in this pathway by repressing both *retS* and *rsmZ* (Figure 7A). Analysis of the *rsmZ* promoter revealed a candidate PhoP binding site, between the start codon and the −10 promoter region (Figure 8A), similar to the PhoP-repressing binding site identified in the *retS* promoter (Figure 7B). Binding assays indicated that His_6_-PhoP was capable of direct binding to the *rsmZ* promoter (Figure 8B). Taken together, these data indicate that Mg^2+^ limitation is sensed by the PhoPQ two-component system, which acts as a direct negative regulator of both *retS* and *rsmZ* expression, resulting in enhanced biofilm formation.

## Discussion

Previous studies of *P. aeruginosa* have identified multiple signaling networks that control biofilm formation. These include the sensor kinases GacS, RetS and LadS that comprise a sophisticated regulatory network involved in reciprocal regulation of biofilm-associated and classical virulence-associated genes [19], [20]. However, the central question regarding the role of TCS in biofilm formation remains the identification of the actual environmental signals that are sensed.

The gene expression profiling approach described here, successfully identified Mg^2+^ limitation as an environmental condition that simultaneously repressed *retS* expression and induced expression of the *pel/psl* EPS biosynthesis genes. Our observation of transcriptional effects on the EPS biosynthesis genes that correlated with decreased levels of *retS* expression is consistent with a previous microarray study that detected transcriptional regulation of *pel* and *psl* in a *retS* mutant [19]. An additional study has identified post-transcriptional regulation of *psl* through RsmA [26], which lies downstream of RetS in the regulatory pathway. Thus, RetS is capable of regulating EPS production by both transcriptional and post-transcriptional mechanisms.

The kinetics of gene expression in Figure 2 revealed three gene expression patterns during the early, middle and late stages of growth. The known PmrA- and PmrA/PhoP-regulated genes *PA4774 and PA3553* (*arnC*) were induced early in growth at 1.5 and 5 h, respectively. The exclusively PhoP-controlled gene *oprH* was maximally induced at the middle stage of growth, 13.5 h. Maximal induction of *rsmZ*, *rsmY*, *pel* and *psl* occurred at later stages of growth following repression of *retS* between 7.5 and 14 h (Figure 2A). Other sensor/regulator genes tested but which did not show a significant fold change under high and limiting Mg^2+^ conditions included *rocR*, *rocA1*, *ladS*, *gacS*, *fleSR* and *pilSR*. In agreement with the gene expression profile, growth under Mg^2+^ limitation caused a switch to the biofilm mode with increased aggregation (Figure 3), EPS production (Figure 5) and a hyperbiofilm phenotype (Figure 3).

Aggregation effects observed during growth in limiting Mg^2+^ conditions were controlled by PhoP-mediated repression of *retS* and fine-tuning of *rsmZ/rsmY* levels. Analysis of *rsmZ* and *rsmY* expression in *phoP::xylE* indicated that PhoP acts differently on the expression of these two sRNA-encoding genes, directly controlling the expression of *rsmZ* and indirectly controlling the levels of *rsmY*. This is similar to previous studies where *rsmY* and *rsmZ* were differentially regulated by other TCS [55], [56] and further suggests that *rsmZ* and *rsmY* are not functionally redundant but may exert different effects depending on the environmental conditions. In the wildtype PAO1, both *rsmZ* and *rsmY* are induced under Mg^2+^ limitation (Figure 2). This is likely a consequence of reduced RetS levels and activation of the GacAS pathway [19]. However the relative level of *rsmY* in PAO1 is significantly higher compared to that of *rsmZ* (45-fold) (Figure 7A), suggesting that expression of *rsmZ* is limited by PhoP repression (Figure 8). It may be that direct PhoP regulation of *rsmZ* serves to fine-tune the relative levels of the small regulatory RNAs, in order to maintain higher relative levels of *rsmY*, which corresponds to increased biofilm formation. This is consistent with a recent report showing that the overexpression of *rsmZ*, but not *rsmY*, impairs *P. aeruginosa* biofilm formation [56].

Extracellular DNA has also been shown to be important for the early stages of biofilm formation [37] and Mg^2+^ sequestration by DNA may play a role in maturation of *P. aeruginosa* biofilms by inducing EPS matrix production. Extracellular DNA, of both bacterial and eukaryotic origin, can be found at high concentrations (up to 20 mg/ml) in the CF lung [33], [35], [36]. DNA also accumulates in other environmental niches of *P. aeruginosa*, including soil ([57] and aquatic environments [34], and therefore likely contributes to *P. aeruginosa* biofilm formation in the natural environment.

Cation chelation by both DNA and EDTA can induce biofilm formation (Figure 4B). This observation is in contrast to a previous study, which showed that EDTA is a potent biofilm disrupter in *P. aeruginosa* [58]. This apparent contradiction can be explained by opposite effects of subinhibitory and lethal concentrations of chelators. Since EDTA is lethal at a concentration of 1 mM by disrupting membrane integrity [31], it is not surprising that 50 mM EDTA disrupts the biofilm matrix structure and kills biofilm cells [58].

This study identifies Mg^2+^ limitation as an important environmental trigger of *P. aeruginosa* biofilm development and increases our understanding of the potential role for extracellular DNA and cation chelation in the regulation of antibiotic resistant and aggregative biofilms. Novel approaches to block the PhoPQ and/or RetS signaling pathways, or to neutralize extracellular DNA, may be an effective and novel treatment strategy to prevent or reduce biofilm formation during infections and in the natural environment.

## Materials and Methods

### Strains, plasmids and media conditions

All strains and plasmids are listed in Tables 1 and 2, respectively. For details on construction of promoter fusions see Methods S1 primer Table S2. *P. aeruginosa* strains were routinely grown and maintained on Luria-Bertani (LB) plates or LB broth at 37°C and cultured in defined basal medium 2 (BM2) media [59] containing 0.02 mM Mg^2+^ (limiting) and 2 mM Mg^2+^ (high). Trimethoprim (Sigma-Aldrich) was added at 300 µg/ml for selection and 150 µg/ml for plasmid maintenance. Unless otherwise stated succinate (20 mM) was used as the carbon source. The source of DNA was fish sperm DNA-potassium salt (USB, Cleveland, OH).

**Table 1:** Strains used in this study.

Strain name	Description or Mutant ID	Reference
PAO1	Wild-type *P. aeruginosa* PAO1	[38]
PAO1w	Wozniak lab wild-type PAO1	
PAO1 pCHAP6656	PAO1 carrying pCHAP6656	[62]
*phoP*::*xylE*	*phoP* mutant (*phoP*::*xylE-aacC1*)	[63]
*pelA::lux*	44_G8 (*pelA* mutant)	[64]
*pelB::lux*	66_B7 (*pelB* mutant and transcriptional *lux* fusion)	[64]
*pelD::lux*	53_C4 (*pelD* mutant and transcriptional fusion)	[64]
*PA4774::lux*	11_A2 (*PA4774* mutant and transcriptional *lux* fusion)	[64]
*PA3553::lux*	53_D10 (*PA3553* mutant and transcriptional *lux* fusion)	[64]
*PAZH13*	*rsmA* mutant	[65]
*retS::lux*	18_F4 (*retS* mutant)	[64]
*pslH-*	*pslH* mutant (WFPA818)	[43]
*pel/psl-*	Double *pel/psl* mutant	D. Wozniak (unpublished)
PAO1 *xcpR::lux*	*xcpR* promoter *lux* fusion integrated at *attB* site	[66]
PAO1 *aprA::lux*	*aprA* promoter *lux* fusion integrated at *attB* site	[66]
PAO1 *oprH::lux*	*orpH* promoter *lux* fusion integrated at *attB* site	[66]
PAO1 *exoT::lux*	*exoT* promoter *lux* fusion integrated at *attB* site	[66]

**Table 2:** Plasmids used in this study.

Plasmids	Description	Reference
pMS402	Expression reporter plasmid carrying the promoterless *luxCDABE* genes	[67]
pMS402*retS*-*lux*	pMS402 containing *retS* promoter	This study
pMS402*pslA*-*lux*	pMS402 containing *pslA* promoter	This study
pMS402*rsmZ*-*lux*	pMS402 containing *rsmZ* promoter	This study
pMS402*rsmY*-*lux*	pMS402 containing *rsmY* promoter	This study
pMS402*ladS*-*lux*	pMS402 containing *ladS* promoter	This study
pMS402*gacS*-*lux*	pMS402 containing *gacS* promoter	This study
pMS402*fleSR*-*lux*	pMS402 containing *fleSR* promoter	This study
pMS402*pilSR*-*lux*	pMS402 containing *pilSR* promoter	This study
pMS402*rocR*-*lux*	pMS402 containing *rocR* promoter	This study
pMS402*rocA1*-*lux*	pMS402 containing *rocA1* promoter	This study
pSCrhaB2	expression vector containing a rhamnose-inducible promoter	[50].
pSCrhaB2RetS	*retS* coding region in pSCrhaB2	This study
p*phoP*-His_6_	His6-PhoP cloned into pET28a	[40].
pMS402*retSm8*-*lux*	pMS402 containing *retS* promoter with ttc to gga substitution	This study
pMS402*retSm13*-*lux*	pMS402 containing *retS* promoter with ttc to cct substitution	This study
pMS402*retSm15*-*lux*	pMS402 containing *retS* promoter with ttc to act substitution	This study

### Gene expression assays

Gene expression assays were carried out as previously described [31]. Gene expression values (counts per second, CPS) were normalized to cell number (optical density, OD). Gene expression profiles were grouped by hierarchical clustering using complete linkage. Analysis was performed using Cluster 3.0 and visualized using Treeview [60].

### Biofilm, aggregation, congo red and calcofluor binding assays

Biofilm formation was quantified by crystal violet (CV) staining (OD_600_) as previously described [44]. For details see supplementary information. Bright field microscopy was used to assess aggregation in mid-log cultures grown in BM2 media with 2 mM or 0.02 mM Mg^2+^. Congo red binding assays were performed as previously described [43] with minor modifications (see supplementary information). Bacterial aggregates were stained with calcofluor (EPS), 1 µM SYTO9 (live cells) and 10 µM propidium iodide (dead cells and extracellular DNA), mounted on agarose beds. All microscopy was performed using a Leica DMIREB2 inverted microscope equipped with an ORCA-ER digital camera and Openlab software (Improvision) and analysed using Adobe Photoshop.

### Scanning electron microscopy

*P. aeruginosa* peg-adhered biofilms were grown as previously described in high or limiting Mg^2+^ BM2 for 48 h, fixed as previously described [61], gold coated and visualized using a XL30 environmental scanning electron microscope.

### Overexpression of RetS

The coding region of *retS* was PCR amplified, digested with *XbaI* and *HindIII* and ligated into the pSCrhaB2 vector under the control of a rhamnose-inducible promoter (pSCrhaB2RetS) [50]. Biofilms were cultured in BM2 media with 2 mM or 0.02 mM Mg^2+^ in the presence of 0–2% rhamnose.

### PhoP protein purification and gel shift assays

PhoP protein was purified from *Escherichia coli* BL21 (GE Healthcare) containing His_6_-PhoP [40] cloned into pET28a as previously described [40]. A full length *rsmZ* and *retS* and truncated version of the *retS* promoter construct were PCR amplified (primers, Table S2) and digoxigenin (DIG)-labeled using the DIG Gel Shift Kit, 2nd Generation (Roche), according to manufacturer's instructions. Samples were separated by electrophoresis on 6% native polyacrylamide gels. transferred onto nylon membranes, probed with anti-DIG antibodies and chemiluminescence detected on the ChemiDoc XRS system (Bio-Rad).

### Statistical Analysis

Statistical analysis was performed using GraphPad Prism 5 software. 2-way ANOVA was used to calculate significant differences between PAO1 and mutant strains.

## Supporting Information

Figure S1 **Limiting magnesium-induced biofilm formation is dependent on EPS production but independent of pili or flagella production.** Attachment of PAO1 and relevant mutants to polystyrene pegs was assessed by crystal violet staining and OD_600_ measurement in BM2 2 mM Mg^2+^ or 0.02 mM Mg^2+^ at 24 h. Bars represent the average values obtained from eight pegs and the error bars represent the standard deviation. Significant differences were observed between strains grown in BM2 2 mM Mg^2+^ and BM2 0.02 mM Mg^2+^ (a, p<0.05, ANOVA) and between PAO1 and mutant strains grown in BM2 0.02 mM Mg^2+^ (b, p<0.05, ANOVA). PAO1w, (Wozniak laboratory strain, Ohio State University) is the parent strain of the *pslH* and *pel*/*pslH* double mutant.
(TIF)

Figure S2 **EPS mutants grown in BM2 0.02 mM Mg^2+^ failed to aggregate or stain with calcofluor.** Bacteria were grown in BM2 0.02 mM Mg^2+^ supplemented with 200 µg/ml calcofluor (blue, EPS stain). At 24 h cells were removed, stained with 1 µM syto9 (green, live cells) and visualized on agarose beds by fluorescence microscopy. Merged blue/green fluorescence images are representative of three independent experiments.
(TIF)

Figure S3 **PhoP regulates EPS production in** ***P. aeruginosa*** **.** (A) Quantification of EPS production using congo red binding (corrected A490) in PAO1 and *phoP::xylE* grown in BM2 0.02 mM Mg^2+^ at 24 h. (B) Transmission electron microscopy of PAO1 and *phoP::xylE*. Bacteria were grown at 37°C overnight on BM2 0.02 mM Mg^2+^ 0.5% agar plates. Cells were prepared and stained as described by Hyland *et al.* 2006 [61] and examined using a Hitachi S-7000 transmission electron microscope.
(TIF)

Table S1 **List of media used to assess** ***retS*** **expression.**
(DOC)

Table S2 **Primers used in this study.** XhoI restriction sites are bolded; BamHI restriction site are underlined; overlap regions for SOE PCR are bolded and italicized; the modified nucleotides of the PhoP box in the retS promoter are capitalized.
(DOC)

Methods S1 **Additional information on strains and methods.**
(DOC)
