# Supplementary figures and images for: Magnesium Limitation Is an Environmental Trigger of the Pseudomonas aeruginosa Biofilm Lifestyle
Source: PLoS One. 2011 Aug 16;6(8):e23307. doi: 10.1371/journal.pone.0023307 (PMC3156716; doi:10.1371/journal.pone.0023307)

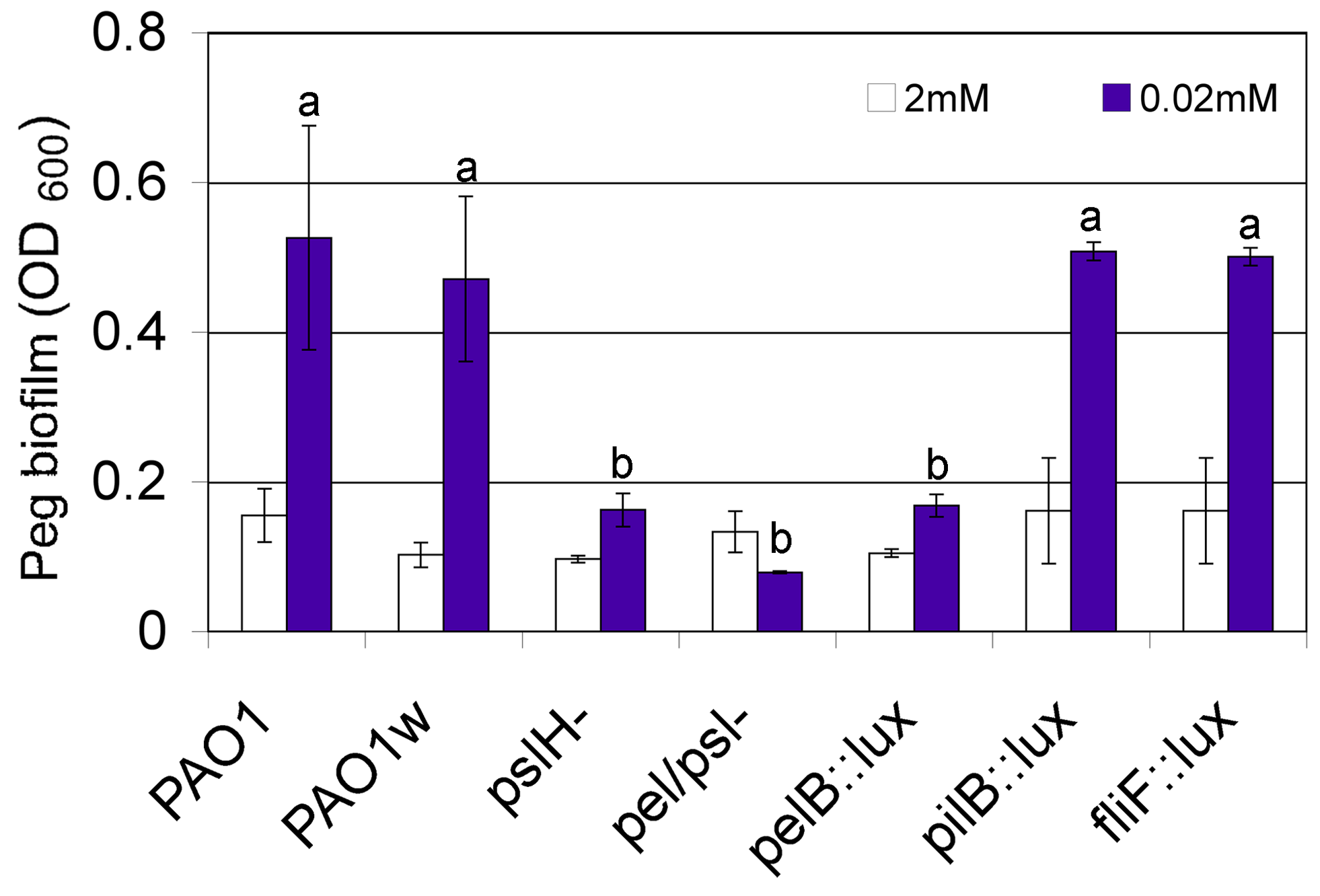

Supplement: Figure S1 — Limiting magnesium-induced biofilm formation is dependent on EPS production but independent of pili or flagella production. Attachment of PAO1 and relevant mutants to polystyrene pegs was assessed by crystal violet staining and OD600 measurement in BM2 2 mM Mg2+ or 0.02 mM Mg2+ at 24 h. Bars represent the average values obtained from eight pegs and the error bars represent the standard deviation. Significant differences were observed between strains grown in BM2 2 mM Mg2+ and BM2 0.02 mM Mg2+ (a, p<0.05, ANOVA) and between PAO1 and mutant strains grown in BM2 0.02 mM Mg2+ (b, p<0.05, ANOVA). PAO1w, (Wozniak laboratory strain, Ohio State University) is the parent strain of the pslH and pel/pslH double mutant. (TIF) [file pone.0023307.s001.tif]

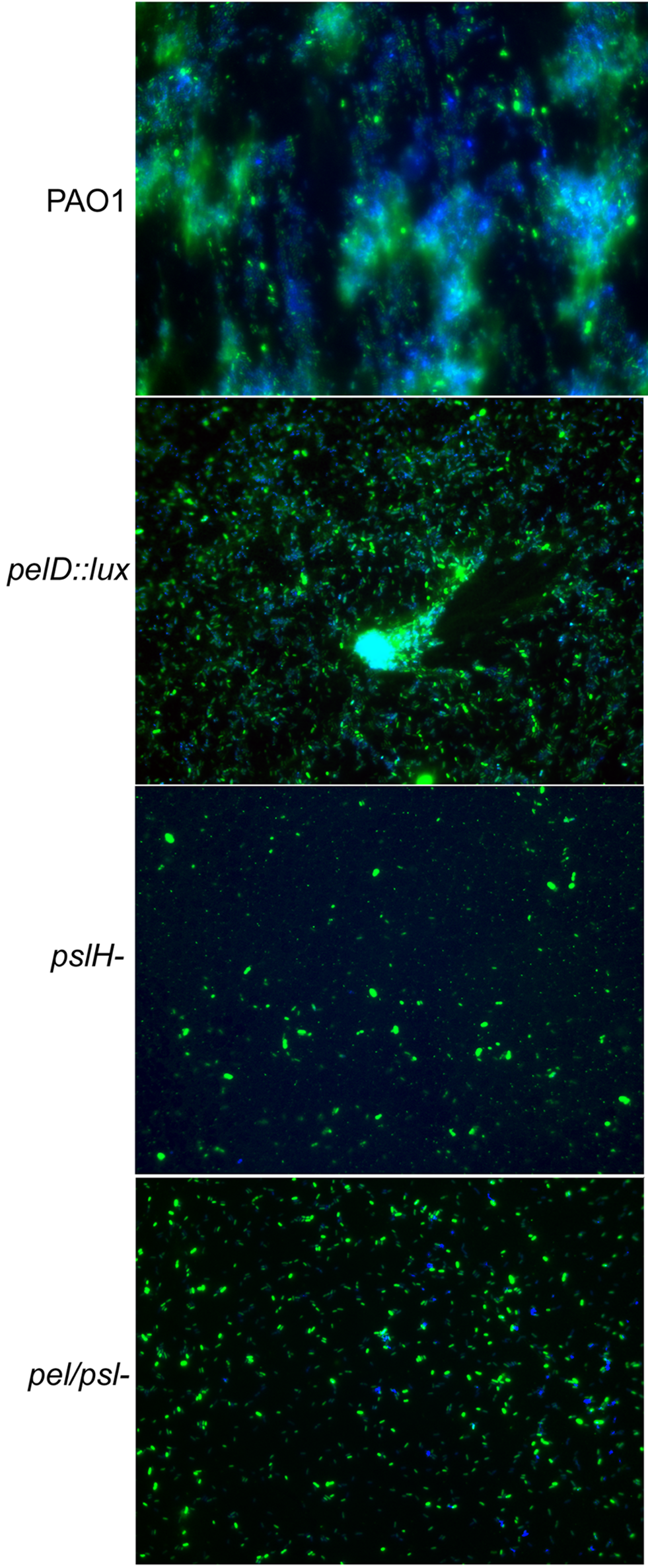

Supplement: Figure S2 — EPS mutants grown in BM2 0.02 mM Mg2+ failed to aggregate or stain with calcofluor. Bacteria were grown in BM2 0.02 mM Mg2+ supplemented with 200 µg/ml calcofluor (blue, EPS stain). At 24 h cells were removed, stained with 1 µM syto9 (green, live cells) and visualized on agarose beds by fluorescence microscopy. Merged blue/green fluorescence images are representative of three independent experiments. (TIF) [file pone.0023307.s002.tif]

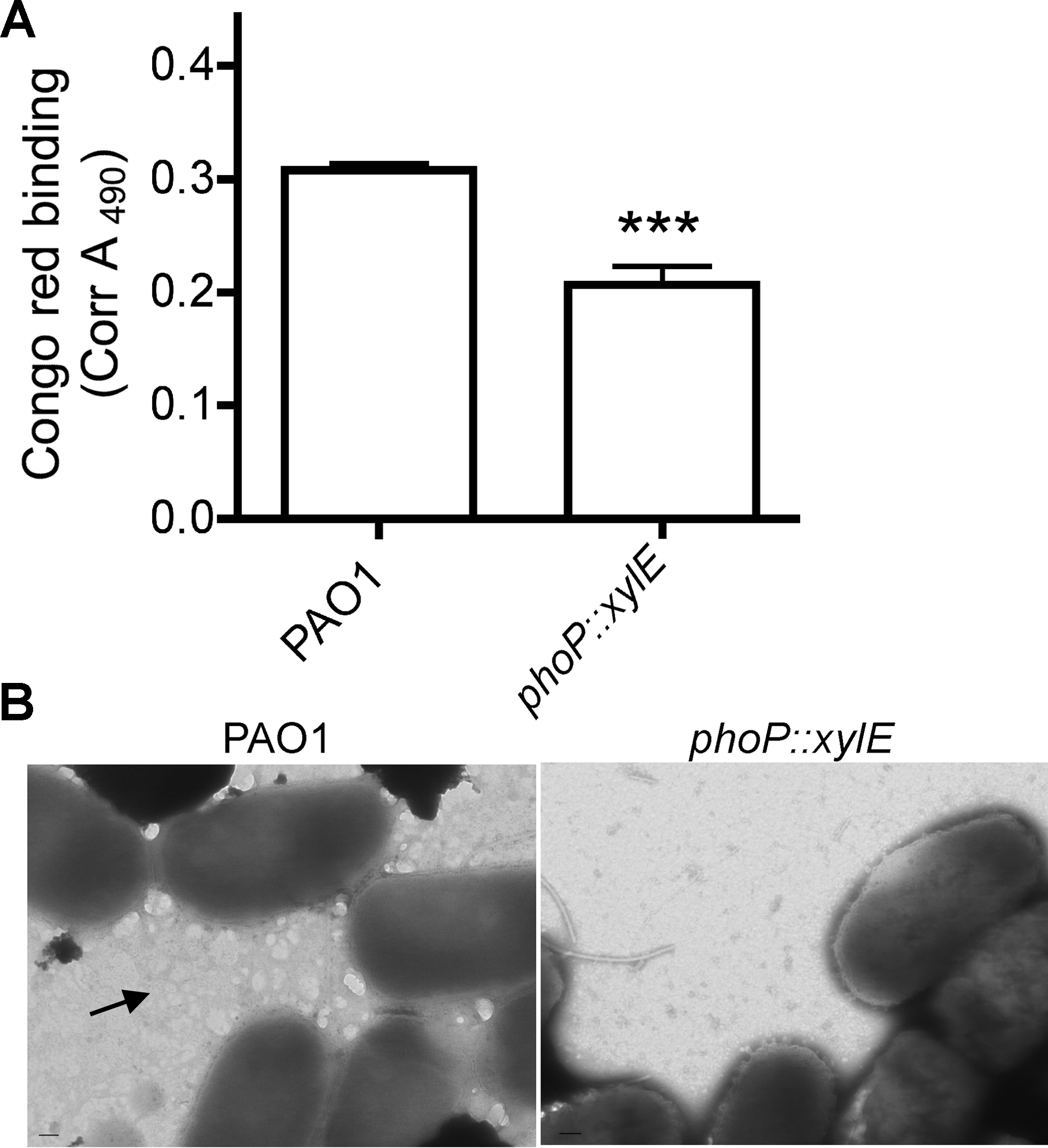

Supplement: Figure S3 — PhoP regulates EPS production in P. aeruginosa . (A) Quantification of EPS production using congo red binding (corrected A490) in PAO1 and phoP::xylE grown in BM2 0.02 mM Mg2+ at 24 h. (B) Transmission electron microscopy of PAO1 and phoP::xylE. Bacteria were grown at 37°C overnight on BM2 0.02 mM Mg2+ 0.5% agar plates. Cells were prepared and stained as described by Hyland et al. 2006 [61] and examined using a Hitachi S-7000 transmission electron microscope. (TIF) [file pone.0023307.s003.tif]
