# Supplementary material for: Magnesium Limitation Is an Environmental Trigger of the Pseudomonas aeruginosa Biofilm Lifestyle
Source: PLoS One. 2011 Aug 16;6(8):e23307. doi: 10.1371/journal.pone.0023307 (PMC3156716; doi:10.1371/journal.pone.0023307)
Supplement: Table S1 — List of media used to assess retS expression. (DOC) [file pone.0023307.s004.doc]

Table S1

| **Media short name** | **Description** | **Reference** |
| --- | --- | --- |
| BM2 | Basal media 2 (2 mM MgSO_4_); carbon source (C is Sodium Succinate unless otherwise stated | [1] |
| BM2 + Ceft | BM2 0.1 μg/ml Ceftazidime |  |
| BM2 + CI | BM2 0.01 μg/ml Ciprofloxacin |  |
| BM2 + pxnB | BM2 0.5 μg/ml of polymyxin B |  |
| BM2 + Tm | BM2 0.1 μg/ml Tobramycin |  |
| BM2 + Gm | BM2 0.3 μg/ml Gentamycin |  |
| BM2 + Pip | BM2 0.5 μg/ml Piperacillin |  |
| BM2 0.3 % agar | BM2 0.3% agar |  |
| LB 30 % sucrose | LB 30% sucrose |  |
| LB 2.5 % NaCl | LB 2.5 % NaCl |  |
| LB 1.5 % NaCl | LB 1.5 % NaCl |  |
| LB 1 % NaCl | LB (salt concentration is routinely 1 % NaCl) |  |
| LB 0.5 % NaCl | LB 0. 5% NaCl |  |
| BM2 0.5 % Mucin | BM2 0.5 % mucin |  |
| BM2 0.5 % NaAlg | BM2 0.5 % sodium alginate |  |
| ASM + | Artifical sputum media + cas amino acids | [2] |
| BM2 C-glucose | BM2 C-20 mM glucose |  |
| BM2 C-citrate | BM2 C-20 mM Sodium Citrate |  |
| BM2 C-0.5 % mucin | BM2 C-0.5 % mucin |  |
| BM2 C-0.5 % EYE | BM2 C- 0.5 % egg yolk emulsion |  |
| BM2 C-0.5 % CAA | BM2 C-0.5 % cas amino acids |  |
| BM2 C-0.5% DNA | BM2 C-0.5 % DNA (fish sperm) |  |
| BM2 0.02 mM Mg | BM2 0.02 mM MgSO_4_ |  |
| BM2 50 μM Fe | BM2 50 μM FeSO_4_ |  |
| BM2 1 μM Fe | BM2 1 μM FeSO_4_ |  |
| BM2 low P | BM2 400 μM P |  |
| LB 2.5 mM CaCl_2_ | LB 2.5 mM CaCl_2_ |  |
| LB 10 mM NTA | LB 10 mM Nitrilotriacetic acid |  |
| 1/4 BHI | 1/4 Brain Heart infusion |  |
| BHI | Brain Heart infusion |  |
| LB | Luria-Bertani |  |

1. Mulcahy H, Charron-Mazenod L, Lewenza S. (2010) *Pseudomonas aeruginosa* produces an extracellular deoxyribonuclease that is required for utilization of DNA as a nutrient source. Environ Microbiol 12(6): 1621-1629.

2. Sriramulu DD, Lunsdorf H, Lam JS, Romling U (2005). Microcolony formation: a novel biofilm model of *Pseudomonas aeruginosa* for the cystic fibrosis lung. J Med Microbiol 54:667-676.
