# Supplementary material for: Magnesium Limitation Is an Environmental Trigger of the Pseudomonas aeruginosa Biofilm Lifestyle
Source: PLoS One. 2011 Aug 16;6(8):e23307. doi: 10.1371/journal.pone.0023307 (PMC3156716; doi:10.1371/journal.pone.0023307)
Supplement: Table S2 — Primers used in this study. XhoI restriction sites are bolded; BamHI restriction site are underlined; overlap regions for SOE PCR are bolded and italicized; the modified nucleotides of the PhoP box in the retS promoter are capitalized. (DOC) [file pone.0023307.s005.doc]

**Table S2.**

| **Gene name** | **Forward** | **Reverse** | **Reference** |
| --- | --- | --- | --- |
| pretS | ga**ctcgag**gcggccgcgtgctctgtg | gaggatccgggaacgctggaaacgccg | [1] |
| prsmZ | gaggatccgccgggttttttgtgtctg | gaggatcccaggagtgatattagcgattc | [2] |
| prsmY | gaggatccggttgaccaggtcgctgct | gaggatccgtcctgacggtttgaagatta | This study |
| ppslA | ga**ctcgag**ccgaaatggcacgaggcggcgctg | gaggatccgccgatcacgggcagtccattga | [3] |
| pladS | gaggatcccggacaggcagagcactacg | gaggatccgacctgctcgttgaagctcac | This study |
| pgacS | gaggatccattgaggtgctggaacagatg | gaggatccccaggtgaagtaaccgccaagc | This study |
| pfleSR | gaggatccgcgcgaagagctggaagagc | gaggatccctggttgaacagcgcgaagg | This study |
| ppilSR | gaggatcccctggtggagctacatgac | gaggatccggtggtacagacggaggatgc | This study |
| procR/rocA1 | gcaggatccgctgtgacggctaccaggc | gcaggatccccatgcttttccagcagg | This study |
| pretS_nophoPR |  | gaggatccgtgaatggccggattatag | This study |
| pretSm8IntF | ***ccttcgaagggac***GGAgccgtggtacgg |  | This study |
| pretSm13IntF | ***ccttcgaagggac***CCTgccgtggtac |  | This study |
| pretSm15IntF | ***ccttcgaagggac***ACTgccgtggtacgg |  | This study |
| pretSIntR |  | ***gtcccttcgaagg***tgaatggccg | This study |

1. Laskowski MA, Osborn E, Kazmierczak BI (2004). A novel sensor kinase-response regulator hybrid regulates type III secretion and is required for virulence in *Pseudomonas aeruginosa*. Mol Microbiol 54:1090-1103.

2. Burrowes E, Abbas A, O'Neill A, Adams C, O'Gara F (2005). Characterisation of the regulatory RNA RsmB from *Pseudomonas aeruginosa* PAO1. Res Microbiol 156:7-16.

3. Overhage J, Schemionek M, Webb JS, Rehm BH (2005). Expression of the psl operon in *Pseudomonas aeruginosa* PAO1 biofilms: PslA performs an essential function in biofilm formation. Appl Environ Microbiol 71:4407-4413.
