# Supplementary material for: Magnesium Limitation Is an Environmental Trigger of the Pseudomonas aeruginosa Biofilm Lifestyle
Source: PLoS One. 2011 Aug 16;6(8):e23307. doi: 10.1371/journal.pone.0023307 (PMC3156716; doi:10.1371/journal.pone.0023307)
Supplement: Methods S1 — Additional information on strains and methods. (DOC) [file pone.0023307.s006.doc]

**Methods S1.**

**Additional strain information**

*Pseudomonas aeruginosa* PAO1 and PAO1w (Wozniak laboratory strain) were used as wildtype strains. The *phoP*::*xylE* mutant was previously constructed [1]. The mini-Tn5-*lux* transposon mutants used in this study (*4774::lux*, *3553::lux*, *pelD::lux, pelA::lux* and *retS::lux*) were previously constructed and mapped [2]. PAO1 strains containing *aprA*, *xcpR*, *oprH* and *exoT* promoters fused to *lux* (luminescence), which are chromosomally integrated were previously described [3]. Plasmid-encoded *lux* reporters to promoters of the *ladS, retS, gacS, gacA, pslA, rsmZ and rsmY* genes were constructed in the plasmid pMS402. For microscopy *P. aeruginosa* pCHAP6656, producing mCherry as an outer membrane-anchored lipoprotein, was used [4].

**Construction of promoter *lux* fusions**

For promoter-*lux* fusions, promoter regions of interest were PCR amplified, digested and cloned into the *BamHI* or the *XhoI-BamHI* site upstream of the *lux* genes on pMS402. Cloned promoter sequences were confirmed by DNA sequencing and transformed into *P. aeruginosa* by electroporation. To construct a *retS* promoter construct that lacked the entire 18 bp PhoP box (truncated retS promoter*)* the retS promoter up to but not including the PhoP box was PCR amplified using primers pretSF and pretS_nophoPR. The PhoP box in *retS* promoter was mutated using a splicing by overlap extension (SOE)-PCR approach [5]. The 5’ promoter region was amplified using primers pretSF and reverse primer pretSIntR. The 3’ promoter region was amplified using pretSR and pretSm8IntF, pretSm13IntF or pretSm15IntF which resulted in a change from TTC in the second PhoP box to GGA, CCT or ACT, respectively. PCR products were purified, diluted 1/100 and a second round of SOE-PCR carried out using primers pretSF and pretSR. PCR products were digested and cloned into the the *XhoI-BamHI* site upstream of the *lux* genes on pMS402.

**Biofilm assays**

LB starter cultures were diluted in the appropriate medium and inoculated at a concentration of 1.5 x 10^6^ cfu/well. Biofilms were cultivated either in shaking microplates (ring biofilms) or on polystyrene pegs (peg biofilms) in shaking microplates or in shaking glass tubes at 37^o^C for 24 h. Under certain growth conditions where very robust biofilms were formed it was found that the biofilms formed on pegs could not be completely solubilized in ethanol. For this reason ring biofilms, which were more easily soluble, were quantified instead of peg-adhered biofilms.

**Congo red and calcofluor binding assays**

For both congo red and calcofluor assays, overnight cultures were grown in LB broth and diluted 1/1000 in BM2 media with 2 mM or 0.02 mM Mg^2+^ containing 40 μg/ml congo red or 200 μg/ml of calcofluor for 24 hours at 37^o^C with agitation. For congo red binding assays after 24 hours of growth, the OD_600_ of the cultures was measured. A 1 ml aliquot was removed and pelleted by centrifugation. For quantification of congo red binding, the OD_490_ of the supernatant of each sample was determined, subtracted from absorbance of the 40 μg/ml congo red standard and normalized to cell number (OD_600_ value). The OD_490_ values indicate the amount of congo red dye bound to the cells.

References

1. Macfarlane EL, Kwasnicka A, Ochs MM, Hancock RE. (1999) PhoP-PhoQ homologues in pseudomonas aeruginosa regulate expression of the outer-membrane protein OprH and polymyxin B resistance. Mol Microbiol 34(2): 305-316.

2. Lewenza S, Falsafi RK, Winsor G, Gooderham WJ, McPhee JB, et al. (2005) Construction of a mini-Tn5-luxCDABE mutant library in pseudomonas aeruginosa PAO1: A tool for identifying differentially regulated genes. Genome Res 15(4): 583-589.

3. Sibley CD, Duan K, Fischer C, Parkins MD, Storey DG, et al. (2008) Discerning the complexity of community interactions using a drosophila model of polymicrobial infections. PLoS Pathog 4(10): e1000184.

4. Lewenza S, Mhlanga MM, Pugsley AP. (2008) Novel inner membrane retention signals in pseudomonas aeruginosa lipoproteins. J Bacteriol 190(18): 6119-6125.

5. Horton RM, Hunt HD, Ho SN, Pullen JK, Pease LR. (1989) Engineering hybrid genes without the use of restriction enzymes: Gene splicing by overlap extension. Gene 77(1): 61-68.
